# An inter-laboratory comparison of standard membrane-feeding assays for evaluation of malaria transmission-blocking vaccines

**DOI:** 10.1186/s12936-016-1515-z

**Published:** 2016-09-09

**Authors:** Kazutoyo Miura, Will J. R. Stone, Karin M. Koolen, Bingbing Deng, Luwen Zhou, Geert-Jan van Gemert, Emily Locke, Merribeth Morin, Teun Bousema, Robert W. Sauerwein, Carole A. Long, Koen J. Dechering

**Affiliations:** 1 Laboratory of Malaria and Vector Research, National Institute of Allergy and Infectious Diseases, National Institutes of Health, 12735 Twinbrook Parkway, Rockville, MD USA; 2 Department of Medical Microbiology, Radboud University Nijmegen Medical Center, 6500 HB Nijmegen, The Netherlands; 3 PATH Malaria Vaccine Initiative, 455 Massachusetts Avenue NW, Washington, DC 20001 USA; 4 TropIQ Health Science, Transistorweg 5, 6534 AT Nijmegen, The Netherlands

**Keywords:** *Plasmodium falciparum*, Gametocyte, Oocyst, Transmission, *Anopheles*, Mosquito, Vaccine, SSM-VIMT

## Abstract

**Background:**

An effective malaria transmission-blocking vaccine may play an important role in malaria elimination efforts, and a robust biological assay is essential for its development. The standard membrane-feeding assay (SMFA) for *Plasmodium falciparum* infection of mosquitoes is considered a “gold standard” assay to measure transmission-blocking activity of test antibodies, and has been utilized widely in both non-clinical and clinical studies. While several studies have discussed the inherent variability of SMFA within a study group, there has been no assessment of inter-laboratory variation. Therefore, there is currently no assurance that SMFA results are comparable between different studies.

**Methods:**

Mouse anti-Pfs25 monoclonal antibody (mAb, 4B7 mAb), rat anti-Pfs48/45 mAb (85RF45.1 mAb) and a human polyclonal antibody (pAb) collected from a malaria-exposed adult were tested at the same concentrations (6–94 μg/mL for 4B7, 1.2–31.3 μg/mL for 85RF45.1 and 23–630 μg/mL for human pAb) in two laboratories following their own standardized SMFA protocols. The mAbs and pAb, previously shown to have strong inhibition activities in the SMFA, were tested at three or four concentrations in two or three independent assays in each laboratory, and percent inhibition in mean oocyst intensity relative to a control in the same feed was determined in each feeding experiment.

**Results:**

Both monoclonal and polyclonal antibodies dose-dependently reduced oocyst intensity in all experiments performed at the two test sites. In both laboratories, the inter-assay variability in percent inhibition in oocyst intensity decreased at higher levels of inhibition, regardless of which antibody was tested. At antibody concentrations that led to a >80 % reduction in oocyst numbers, the inter-laboratory variations were in the same range compared with the inter-assay variation observed within a single laboratory, and the differences in best estimates from multiple feeds between the two laboratories were <5 percentage points.

**Conclusions:**

This study confirms previous reports that the precision of the SMFA increases with increasing percent inhibition. Moreover, the variation between the two laboratories is not greater than the variation observed within a laboratory. The findings of this study provide guidance for comparison of SMFA data from different laboratories.

**Electronic supplementary material:**

The online version of this article (doi:10.1186/s12936-016-1515-z) contains supplementary material, which is available to authorized users.

## Background

Malaria mortality and morbidity have declined significantly in many parts of the world in the last 2 decades [1]. Despite this achievement, the World Health Organization estimated that there were still 438,000 deaths due to malaria in 2015, and that ~70 % of deaths occurred in children less than 5 years old [1]. Recently, Bhatt et al. estimated that 663 million clinical cases have been averted by malaria interventions from 2000 to 2015 in sub-Saharan Africa, largely as a result of insecticide-treated bed nets and indoor residual spraying [2]. However, the emergence of resistance in *Anopheles* vectors to current insecticides, and shifting behavioural patterns among vector groups are a great concern for future malaria control [3]. In addition, the spread of resistance to artemisinin-based combination therapy among Southeast Asian parasite strains have been reported [4]. Novel interventions are, therefore, important extensions to the current range of control tools, and may be necessary to achieve elimination in many currently endemic areas [5]. Transmission-blocking vaccines that interrupt human-to-mosquito transmission by targeting the sexual, sporogonic, or mosquito stages of the parasite are called SSM-VIMT. SSM-VIMT have the potential to reduce malaria transmission from humans to mosquitoes for whole populations, and could be an important supplement to traditional controls in countries striving for malaria elimination [6–8].

SSM-VIMT are designed to elicit anti-parasite or anti-mosquito antibodies in vaccinees, and the antibodies block parasite development in the mosquito vector when ingested with gametocytes: the sexual-stage, transmissible form of the malaria parasite. While several different assays can be applied for SSM-VIMT development [9], the standard membrane-feeding assay (SMFA) is considered the “gold standard” for determining the impact of test factors on gametocyte infectivity to mosquitoes (either measured by a reduction in oocyst intensity or in prevalence of infected mosquitoes). The SMFA has broad utility, and has been employed to evaluate the functionality of vaccine or whole-parasite induced antibodies in animal studies and human clinical trials [7, 8, 10–12], as well as antibodies induced by natural exposure to malaria infection in endemic settings [13–16]. Furthermore, increasing interest in transmission-blocking drugs has made the SMFA a useful assay for malaria drug development [17–19].

While there are variations in SMFA methodology among different investigators, the assay is generally conducted by feeding a blood meal containing a mixture of cultured *Plasmodium falciparum* gametocytes and test (or control) antibodies to *Anopheles* mosquitoes through a membrane-feeding apparatus. Approximately 1 week later mosquitoes from the test and control groups are examined to enumerate the oocyst-forms of parasites that, if present, can be visualized in the epithelium of the mosquito’s midgut by mercury-bromide staining. A recent study qualified SMFA following the International Conference on Harmonisation (ICH) Harmonised Tripartite Guideline Q2(R1) using an anti-Pfs25 monoclonal antibody (mAb) with a single protocol [20]. The study concluded that the range (the levels of transmission-blocking activity in which the analytical procedure has a suitable level of precision and linearity) of SMFA performed with their method was when there was more than ~80 % inhibition in oocyst intensity. However, there have been no direct studies, which assess inter-laboratory variation in % inhibition in SMFA.

Modern vaccine development relies heavily on collaborative research efforts and product development partnerships that involve multiple laboratories around the world [21]. Though highly standardized SMFAs have been performed in separate facilities, there is no assurance that SMFA data derived from experiments performed by different investigators are comparable. To enable such comparison, SMFA performance using the same test antibodies was assessed at two laboratories, TropIQ Health Sciences (TropIQ, using methodologies adapted from Radboud University Medical Center [Radboudumc], Nijmegen, The Netherlands) and the Laboratory of Malaria and Vector Research (LMVR, USA). Variation between the laboratories was assessed using a mouse mAb, a rat mAb, and human polyclonal antibody (pAb), all tested at several concentrations in two or three independent assays. When conducted under controlled conditions in different laboratories, the SMFA can provide informative comparable data for SSM-VIMT development.

## Methods

### Test materials

The details of mouse 4B7 (anti-Pfs25) mAb [22] and rat 85RF45.1 (anti-Pfs48/45) mAb [23] used in this study have been described previously. A protein G purified normal mouse IgG was used as the negative control for SMFA with 4B7 and 85RF45.1 mAbs, and the details of IgG preparation were described previously [20]. Human serum was sourced from a malaria-exposed Dutch expatriate with well documented antibody-mediated transmission-blocking activity, who gave verbal, but not written informed consent for his blood sample to be used for malaria research [24]. A pool of negative human sera was obtained from malaria-naive Dutch adults at Sanquin blood bank (Nijmegen, the Netherlands). From the positive and negative human sera, total IgGs were purified according to the manufacturer’s instructions using a GE Healthcare AB Spintrap purification kit (Little Chalfont, United Kingdom) at TropIQ/Radboudumc.

### SMFA

The standardized methodology for performing the SMFA at LMVR has been described previously [20]. The methodology at TropIQ has also been described elsewhere and is based on that developed at Radboudumc and uses mosquitoes from Radboudumc facilities [25]. The TropIQ method differs from the Radboudumc method in that the quality of the gametocyte culture is assessed based on microscopic examination of male exflagellation instead of determination of ookinete formation in a sample that is fed to mosquitoes a day before the actual feed. In addition, the TropIQ method uses a different pipetting scheme that uses pooled reagents/parasites where possible as opposed to preparing each blood meal individually. The differences between the LMVR and TropIQ assays are summarized in Table 1. In this study, all SMFAs were conducted with *P. falciparum* NF54 line parasites and *Anopheles stephensi* mosquitoes that were propagated independently in the two laboratories. Oocyst counts were performed microscopically. Throughout the text, antibody concentration is expressed relative to the liquid phase in a feeder, i.e., serum and buffer, and does not take into consideration the volume taken up by the red blood cells.

**Table 1:** Details of SMFA methods

Parameters	LMVR	TropIQ	
	All tests	mAbs	Human pAb
Gametocyte culture			
Flask	25 or 75 cm^2^ flask	Tipper culture flask	
Htc^a^ (%)	5.0	5.0	5.0
Feeding			
Total volume (μL)	260	270	1200
Liquid volume (μL)	160	119	696
Liquid contents	100 μL of human serum plus 60 μL of sample in 1xPBS	119 μL of human serum^b^	696 μL of human serum^b^
Htc^c^ (%)	38.5	55.9	42.0
Stage V gametocyte^d^ (%)	0.1–0.2	0.1–1.0	ND^e^
Starving mosquitoes (h)	18–21	1	1
Time of feed (min)	20	10	10
Elimination of unfed mosquitoes from analysis	Only mosquitoes with eggs at the time of dissection are analysed	One hour after feeding, fully fed mosquitoes are selected transported to a bigger cage	
Dissection			
Days post feed (day)	8	8	8
Number of mosquitoes	20	20	30
Quality control			
Pre-feeding	>0.5 % of stage V gametocytaemia and >800/mL of exflagellating centres in the undiluted culture	Positive for male exflagellating gametocytes	
Post-feeding	>4 oocysts/midgut in the assay controls	>70 % infected mosquitoes in the assay controls	

^a^Haematocrit

^b^Add lyophilized human serum to make the liquid volume for test antibody to be 100 % human serum

^c^Final haematocrit in a feeder

^d^Final stage V gametocytaemia in a feeder

^e^Not determined

### Statistical analysis

Two different readouts have been widely used to express SMFA results, % inhibition in mean oocyst intensity (%TRA) and % inhibition in prevalence of infected mosquitoes (%TBA). It was decided to use %TRA for this analysis, since %TBA is dependent on the mean oocyst count in the control mosquitoes, and can only be reasonably estimated from the control mean and %TRA [26]. %TRA was calculated as: 100 × {1 − (mean number of oocysts in the test group)/(mean number of oocysts in the control groups)}. Percent inhibition of a test sample was always calculated against a control sample (either normal mouse antibody or normal human antibody) examined in the same feeding experiment. In each laboratory, the same samples were tested in 2 or 3 independent assays at the same concentrations. The best estimate and 95 % confidence intervals (95 % CIs) of %TRA from the multiple feeding experiments for each test antibody at each concentration in each laboratory were calculated using a negative binomial model with zero inflation model, as described previously [20]. To examine inter-laboratory variability, a multiple linear regression was utilized. Previous studies have shown that when the dose of antibody is plotted on a square root scale against inhibitory activity, specifically log-transformed mean oocyst ratio between test and control, SMFA data can be approximated to a linear regression [11, 20, 27]. Therefore, the same transformation was performed for the linear regression analysis. Since 100 %TRA data points were outside of linear range (i.e., log-transformed values became infinity), such points were excluded. The dose of antibody, laboratory of performing SMFA and the interaction of the two were used as explanatory variables. For the IC_50_ estimation, logistic regression was performed as described [19]. Briefly, oocyst counts from individual mosquitoes exposed to varying concentrations of test antibody were fitted to a Hill equation using Maximum Likelihood Estimation to find the best fit. All statistical tests were performed using R (R Foundation for Statistical Computing, Vienna, Austria), JMP12 (SAS Institute, Inc., Cary, NC) and p values <0.05 were considered significant.

## Results

### Similar correlations between mean oocysts and standard deviations or prevalence between the two laboratories

**Fig. 1:**
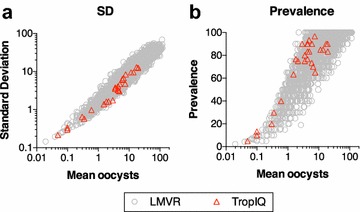
Strong correlations between mean oocysts and the standard deviation or the prevalence at both LMVR and TropIQ. For each COM, mean number of oocysts, the standard deviation **a** and the prevalence of infected mosquitoes **b** were calculated. Each point represents the data from a single COM. LMVR data include all COMs examined at LMVR in the last 5 years (n = 3014), and TropIQ data include only COMs used in this study (n = 33)

Several groups have reported that the distribution of oocyst numbers is explained well by a (zero-inflated) negative binomial model [20, 25, 28, 29]. TropIQ and LMVR use slightly different SMFA protocols (Table 1). To assess whether the data from the two laboratories show an overall similar distribution, the correlations: (1) between the mean number of oocysts in the mosquito and the standard deviation (SD) of the mean oocyst number, and (2) between mean oocyst number and the prevalence of infected mosquitoes (the fraction of mosquitoes containing any number of oocysts) were evaluated. For this purpose, LMVR historical data (including data generated outside of the present study) and TropIQ data (generated in the present study) were compared. For each “Container of Mosquitoes” (COM), the mean oocysts, the SD and the prevalence of infected mosquitoes were calculated. Throughout the paper, COM refers to a group of mosquitoes (~20 to 30 mosquitoes), which were housed in the same container and were fed the using same feeder with the same final mixture of gametocyte cultures and control/test antibodies. As shown in Fig. 1, the correlations observed in the TropIQ data generated in this study overlap with those observed in LMVR historical data. As there are no indications for a divergent data structure, the zero-inflated negative binomial model previously described by LMVR [20] was used to analyse the data sets from both laboratories. Interestingly, a similar correlation between mean oocysts and prevalence was also observed in SMFA with *Plasmodium berghei* parasites [29].

### SMFA comparison with mAbs

**Fig. 2:**
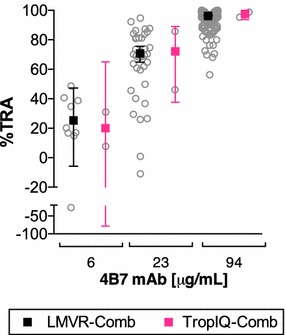
SMFA comparison with 4B7 mAb. In LMVR, mouse 4B7 mAb was tested at 6 (n = 9), 23 (n = 35) and 94 (n = 181) μg/mL in multiple feeding experiments. At TropIQ, 4B7 mAb was tested at the same concentrations in two independent feeding assays. Individual feeding data and the best estimate of %TRA (with the 95 % CIs) from multiple feeds (LMVR-Comb or TropIQ-Comb) are shown. Average oocysts in a negative control antibody (normal mouse IgG) of TropIQ-feed 1(F1) and -feed 2(F2) experiments were 6.5 and 4.2, respectively. Average oocysts in negative control antibodies for LMVR experiments was 21.6 (SD = 18.5)

A mouse 4B7 (anti-Pfs25) mAb, which had been tested in many independent assays at LMVR, was tested at TropIQ at three different concentrations in two independent assays (Fig. 2). Inter-assay variation was calculated as the variation between different assay repeats of a single antibody condition within each laboratory, while inter-laboratory variation is given as the difference in inhibition for all assay repeats of a given condition between laboratories. At LMVR, the best estimates and the 95 % CIs of %TRA by 4B7 mAb were 25 (95 % CI −6 to 47: n = 9), 71 (65 to 76: n = 35) and 96 (96 to 97: n = 181) at 6, 23 and 94 μg/mL, respectively. The best estimates and the 95 % CI of TropIQ data were 20 (−76 to 65), 72 (38 to 89) and 98 (94 to 99) at 6, 23 and 94 μg/mL, respectively (n = 2 at each concentration). In agreement with previous work [20], inter-assay variation decreased with increasing activity of the antibody, as indicated by the smaller confidence intervals at higher %TRA, in both laboratories. Since 4B7 mAb was tested in many more feeding experiments at LMVR, the 95 % CIs at LMVR were smaller. In addition, fewer mean oocysts in the controls of the particular TropIQ feeds might partially contribute to the larger 95 % CIs. Nevertheless, the difference between the two laboratories in the best estimate of %TRA was less than 2 % points at 94 μg/mL.

**Fig. 3:**
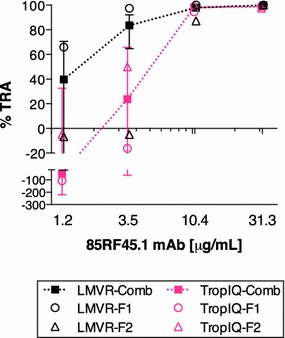
SMFA comparison with 85RF45.1 mAb. In both laboratories, rat 85RF45.1 mAb was tested at 1.2, 3.5, 10.4 and 31.3 μg/mL in two feeding experiments (F1 and F2). The individual data and the best estimate with 95 % CIs from the two feeds (LMVR-Comb or TropIQ-Comb) at each concentration of 85RF45.1 mAb are shown. Average oocysts in a negative control antibody (normal mouse IgG) of LMVR-F1, -F2, TropIQ-F1, and -F2 experiments were 7.8, 61.6, 6.5 and 4.2, respectively

Rat 85RF45.1 (anti-Pfs48/45) mAb was next tested at four different concentrations in two independent assays in both laboratories. The mAb dose-dependently inhibited oocyst formation. At higher concentrations of the mAb (i.e. 10.4 and 31.3 μg/mL), the %TRA observed for replicate experiments within labs and between labs were very comparable (Fig. 3). At lower concentrations, the observed %TRA diverged more between replicate experiments, both within and between laboratories (i.e. concentrations of 1.2 and 3.5 μg/mL). Since four different doses of mAb were tested in each feed, and the same number of feeds were conducted in each laboratory, further analysis was performed to determine the effects of dose and laboratory on inhibition. In a multiple linear regression analysis, dose of 85RF45.1 mAb, laboratory, and the interaction of the two were used as explanatory variables. The overall fit to the linear regression model was R^2^ = 0.73. While there was a significant effect of dose (p = 0.032), the laboratory (p = 0.427) and interaction term (dose and laboratory, p = 0.659) had no significant impact. The effect of dose on oocyst intensity was further investigated by fitting the data to a Hill equation (see Additional file 1). Resulting IC_50_ estimates (the concentration at which the mAb shows 50 % inhibition) were 1.2 and 8.8 µg/mL [feed 1 (F1) and feed 2 (F2) experiments] for data generated at LMVR and 3.4 and 2.5 µg/mL (F1 and F2) for data generated at TropIQ.

### SMFA comparison with human polyclonal antibody

**Fig. 4:**
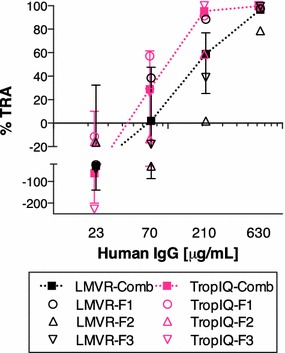
SMFA comparison with positive human pAb. In both laboratories, test human pAb was tested at 23, 70, 10.4, 210 and 630 μg/mL in three feeding experiments (F1, F2 and F3). The individual data and the best estimate with 95 % CIs from the three feeds (LMVR-Comb or TropIQ-Comb) at each concentration of test human pAb are shown. Average oocysts in the negative control (normal human IgG) of LMVR-F1, -F2, -F3, TropIQ-F1, -F2 and -F3 experiments were 3.9, 60.3, 14.0, 16.9, 4.3 and 5.9, respectively

Lastly, a human pAb, which has shown a strong inhibitory activity in SMFA, was tested at four concentrations (23, 70, 210 and 630 μg/mL) in three independent assays in each laboratory (Fig. 4). At 630 μg/mL, the pAb showed a strong and reproducible  %TRA in both laboratories (best estimate of 96.6 %TRA at LMVR and 99.6 at TropIQ), and there was a dose-dependent reduction in oocyst numbers observed in each feeding experiment. As with the experiments using the rodent mAbs, inter-assay variation was larger at lower inhibitions. When a multiple linear regression analysis was performed using all four pAb concentrations (overall fit was R^2^ = 0.79), there was a significant effect of dose (p < 0.0001) on inhibition, but the effect of the laboratory (p = 0.232) and interaction term (dose and laboratory, p = 0.206) were not significant. Fitting the data to a Hill equation revealed IC_50_ estimates of 126, 329 and 158 μg/mL at LMVR, and 64, 66 and 198 μg/mL at TropIQ (see Additional file 2).

## Discussion

This is the first study to evaluate inter-laboratory variation of the SMFA, the “gold standard” assay for *Plasmodium* gametocyte infectivity, using a range of transmission reducing test antibodies (mouse and rat mAbs, and human pAb). In this study, regardless of antibody type or the targets recognized by the antibodies tested, inter-assay and inter-laboratory variation decreased with increasing inhibition of oocyst formation. Conversely, variation became larger in both laboratories with decreasing percent inhibition. These results are in line with previous observations that precision of the SMFA increases with higher percentage reduction, and that levels of inhibition below 80 % need to be interpreted with caution when low numbers of replicate data are available [29]. Across the range of antibodies and concentration tested, inter-laboratory variation did not appear to be greater than the inter-assay variation observed within a laboratory. However, inter-assay variations were larger at lower levels of inhibition, therefore, it is possible that inter-laboratory variations could not be detected, if any, in the lower range. Since the error in %TRA estimate changes gradually (larger error with lower %TRA) as expected from the zero-inflated negative binomial model [20] and shown in this study, it may be difficult to establish a specific level of %TRA at which the inter-assay or inter-laboratory variations are acceptable in all situations. For example, in assessing samples from a clinical study it may be justifiable to set up more stringent criteria than that in a novel candidate discovery study. However, this study showed that at antibody concentrations that led to a >80 % reduction in oocyst numbers, the differences in best estimates of %TRA from multiple feeds between the two laboratories were less than 5 % points.

The SMFA is critical to SSM-VIMT development and there are preceding studies that provide several guidances to optimize intra-laboratory precision when performing SMFA. Medley et al. suggested that at least 50 mosquitoes (ideally 100) need to be dissected to accurately assess transmission-blocking activity [28]. Another study by van der Kolk et al. concluded that if all samples are not tested in a single experiment, data from different assays should be compared only when the mean intensity in the control is at least 35 oocysts per mosquito [13]. Churcher et al. simulated how many mosquitoes were required for dissection per feed to ensure that a reported percent inhibition value had within 10 % error from the true efficacy [29]. For example, if the mean oocyst number in the control is 5, it is estimated that ~200 mosquitoes need to be dissected to accurately report 80 % inhibition, whereas if the mean oocyst number is 100, approximately 50 mosquitoes need to be dissected.

At the present time, it is practically challenging and resource intensive to only regard SMFA experiments with a mean control oocyst intensity of 35 oocysts per mosquito as valid. In terms of the mosquito supply and maintenance, amount of test material required, and the labor intensiveness of dissection, it is also impractical to routinely perform the SMFA with 200 mosquitoes per COM (to be ready if the mean oocysts number in the control were to be 5 oocysts), To some degree, these difficulties can be overcome using one of several modified methods recently published [19, 25, 30], i.e. higher throughput SMFA using transgenic parasites that allow (semi-) automated detection of infection and may eliminate the need for mosquito dissection. However, the modified assays are not necessarily applied to all laboratories at present. Therefore, the comparison study was conducted using the SMFA methods usually performed in many laboratories; i.e. by dissection of 20–30 mosquitoes per feed. With these study conditions, SMFA showed high precision only at higher levels of inhibition. The modifications of the assay discussed above (e.g., dissecting 200 mosquitoes per COM, only utilizing SMFA data when mean control oocyst intensity is >35) should expand the range of % inhibition where the precision of the assay is acceptable. However, application of such modifications for every single assay is impractical at present. The aim of this study was to evaluate the inter-assay and inter-laboratory variations using methods which can be applied in many laboratories routinely. The minor differences between the two laboratories in methods did not cause any measurable “laboratory” effect on percent inhibitions. The present work stems from observations from a preliminary study where results with 4B7 mAb from two additional laboratories were compared. While there was an unfortunate loss in stability of the antibody during preparation and shipment, the results showed similar trends in percent inhibition between the labs despite several differences in methodology (Yimin Wu, Rhoel Dinglasan, personal communication). The approach used here will be useful to determine whether any difference in SMFA procedure affects SMFA readouts.

Because %TRA plateau at low and high concentrations of the test antibody (which gives 0 %TRA and 100 %TRA, respectively), the SMFA cannot discriminate between doses that fall within these upper or lower plateaus. The results of this study indicate that precision of the SMFA is very high at (near-) saturated antibody concentrations, where the sensitivity to changes in antibody concentrations is low. To reveal any differences in assay sensitivity between the two laboratories, IC_50_ values observed for the rat 85RF45.1 and human polyclonal samples were compared. For all antibodies tested here, the dose–effect relationship fitted well to a Hill equation, concordant with the notion that the interaction between an antibody and a ligand can be described by equilibrium kinetics [31]. The differences in IC_50_ values between laboratories were not larger than the inter-assay differences observed within a single laboratory. The SMFA data from this study also fitted well with a linear regression when doses and %TRA were transformed appropriately [11]. While the dose effect was significant, the test site did not significantly influence the outcome. Compared to analyses that evaluate single doses, the full dose response analyses takes into account all data points. This increase in number of observations is likely to increase precision of the measure of inhibition. Moreover, an understanding of the dose–effect relationship will guide predictions of the minimally required titer to achieve the desired reduction in transmission in human SSM-VIMT studies and campaigns. However, to demine the dose–effect relationship, each test sample needs to be tested at multiple concentrations. Therefore, the best balance between throughput of assay and precision/sensitivity needs to be optimized on a per study basis.

This study showed large variation in baseline oocyst intensities between different feeds (ranging from 4 to 62 mean oocysts in this study). Standardization of the average number of oocysts in assay controls would benefit from a gametocyte fertility marker, which is lacking at the moment. Nevertheless, the data indicate that the relative reduction in oocyst intensity (%TRA) can be measured reproducibly at higher levels of inhibition when oocyst intensity in the controls differed between 4 and 62. A recent study conducted at LMVR (repeat assays with 4B7 mAb and simulations using a zero-inflated negative binomial model) also supports that %TRA is independent from control oocyst intensity in SMFA (mean control oocyst intensity in the study ranged from 0.1 to 73.7) [26]. This study reconfirms that an inter-assay variability in the SMFA decreases with increasing percentage inhibition and that variability was demonstrated to be similar between different laboratories. While a weaker inhibitory activity reported from any laboratory needs to be interpreted carefully (unless tested many times or with many mosquitoes), results are very reproducible in higher percentage inhibition. The SMFAs that are performed at the two test sites described here give similar results in terms of precision and sensitivity. This is important for the screening and development of transmission reducing antibodies or compounds, as it alleviates the necessity of performing head-to-head analyses in the same assay(s) at the same laboratory, and thereby increases the flexibility in multi-center vaccine development programs. While additional tests are likely to be required when other laboratories participate in multi-centre SMFA evaluations, the study design and findings presented in this manuscript will provide guidance for the additional testing, thus supporting future SSM-VIMT development.

## Additional files

10.1186/s12936-016-1515-z IC50 calculations for 85RF45.1 mAb.
10.1186/s12936-016-1515-z IC50 calculations for positive human pAb.

## Abbreviations

SMFA: standard membrane-feeding assay
mAb: monoclonal antibody
pAb: polyclonal antibody
SSM-VIMT: transmission-blocking vaccines that interrupt human-to-mosquito transmission by targeting the sexual, sporogonic, or mosquito stages of the parasite
Radboudumc: Radboud University Medical Center
LMVR: Laboratory of Malaria and Vector Research
%TRA: percent transmission reducing activity
COM: container of mosquitoes

## References

[1] WHO. World Malaria Report 2015. Geneva: World Health Organization. 2015. http://www.who.int/malaria/publications/world-malaria-report-2015/report/en/. Accessed 06 May 2016.

[2] Bhatt S, Weiss DJ, Cameron E, Bisanzio D, Mappin B, Dalrymple U (2015). The effect of malaria control on *Plasmodium falciparum* in Africa between 2000 and 2015. Nature.

[3] White NJ, Pukrittayakamee S, Hien TT, Faiz MA, Mokuolu OA, Dondorp AM (2014). Malaria. Lancet..

[4] Ashley EA, Dhorda M, Fairhurst RM, Amaratunga C, Lim P, Suon S (2014). Spread of artemisinin resistance in *Plasmodium falciparum* malaria. N Eng J Med..

[5] Griffin JT, Hollingsworth TD, Okell LC, Churcher TS, White M, Hinsley W (2010). Reducing *Plasmodium falciparum* malaria transmission in Africa: a model-based evaluation of intervention strategies. PLoS Med..

[6] malERA Consultative Group on Vaccines (2011). A research agenda for malaria eradication: vaccines. PLoS Med..

[7] Wu Y, Sinden RE, Churcher TS, Tsuboi T, Yusibov V (2015). Development of malaria transmission-blocking vaccines: from concept to product. Adv Parasitol.

[8] Nikolaeva D, Draper SJ, Biswas S (2015). Toward the development of effective transmission-blocking vaccines for malaria. Expert Rev Vaccines.

[9] Sinden RE, Blagborough AM, Churcher T, Ramakrishnan C, Biswas S, Delves MJ (2012). The design and interpretation of laboratory assays measuring mosquito transmission of *Plasmodium*. Trends Parasitol.

[10] Kapulu MC, Da DF, Miura K, Li Y, Blagborough AM, Churcher TS (2015). Comparative assessment of transmission-blocking vaccine candidates against *Plasmodium falciparum*. Sci Rep..

[11] Miura K, Takashima E, Deng B, Tullo G, Diouf A, Moretz SE (2013). Functional comparison of *Plasmodium falciparum* transmission-blocking vaccine candidates by the standard membrane-feeding assay. Infect Immun.

[12] Wu Y, Ellis RD, Shaffer D, Fontes E, Malkin EM, Mahanty S (2008). Phase 1 trial of malaria transmission blocking vaccine candidates Pfs25 and Pvs25 formulated with montanide ISA 51. PLoS ONE.

[13] van der Kolk M, De Vlas SJ, Saul A, van de Vegte-Bolmer M, Eling WM, Sauerwein RW (2005). Evaluation of the standard membrane feeding assay (SMFA) for the determination of malaria transmission-reducing activity using empirical data. Parasitology.

[14] Drakeley CJ, Bousema JT, Akim NI, Teelen K, Roeffen W, Lensen AH (2006). Transmission-reducing immunity is inversely related to age in *Plasmodium falciparum* gametocyte carriers. Parasite Immunol.

[15] van der Kolk M, de Vlas SJ, Sauerwein RW (2006). Reduction and enhancement of *Plasmodium falciparum* transmission by endemic human sera. Int J Parasitol.

[16] Bousema T, Roeffen W, Meijerink H, Mwerinde H, Mwakalinga S, van Gemert GJ (2010). The dynamics of naturally acquired immune responses to *Plasmodium falciparum* sexual stage antigens Pfs230 & Pfs48/45 in a low endemic area in Tanzania. PLoS ONE.

[17] Almela MJ, Lozano S, Lelievre J, Colmenarejo G, Coteron JM, Rodrigues J (2015). A new set of chemical starting points with *Plasmodium falciparum* transmission-blocking potential for antimalarial drug discovery. PLoS ONE.

[18] Baragana B, Hallyburton I, Lee MC, Norcross NR, Grimaldi R, Otto TD (2015). A novel multiple-stage antimalarial agent that inhibits protein synthesis. Nature.

[19] Vos MW, Stone WJ, Koolen KM, van Gemert GJ, van Schaijk B, Leroy D (2015). A semi-automated luminescence based standard membrane feeding assay identifies novel small molecules that inhibit transmission of malaria parasites by mosquitoes. Sci Rep..

[20] Miura K, Deng B, Tullo G, Diouf A, Moretz SE, Locke E (2013). Qualification of standard membrane-feeding assay with *Plasmodium falciparum* malaria and potential improvements for future assays. PLoS ONE.

[21] Birkett AJ, Moorthy VS, Loucq C, Chitnis CE, Kaslow DC (2013). Malaria vaccine R&D in the decade of vaccines: breakthroughs, challenges and opportunities. Vaccine..

[22] Barr PJ, Green KM, Gibson HL, Bathurst IC, Quakyi IA, Kaslow DC (1991). Recombinant Pfs25 protein of *Plasmodium falciparum* elicits malaria transmission-blocking immunity in experimental animals. J Exp Med.

[23] Roeffen W, Teelen K, van As J, vd Vegte-Bolmer M, Eling W, Sauerwein R (2001). *Plasmodium falciparum*: production and characterization of rat monoclonal antibodies specific for the sexual-stage Pfs48/45 antigen. Exp Parasitol.

[24] Roeffen W, Mulder B, Teelen K, Bolmer M, Eling W, Targett GA (1996). Association between anti-Pfs48/45 reactivity and *P. falciparum* transmission-blocking activity in sera from Cameroon. Parasite Immunol.

[25] Stone WJ, Churcher TS, Graumans W, van Gemert GJ, Vos MW, Lanke KH (2014). A scalable assessment of *Plasmodium falciparum* transmission in the standard membrane feeding assay using transgenic parasites expressing GFP-luciferase. J Infect Dis.

[26] Miura K, Swihart BJ, Deng B, Zhou L, Pham TP, Diouf A (2016). Transmission-blocking activity is determined by transmission-reducing activity and number of control oocysts in *Plasmodium falciparum* standard membrane-feeding assay. Vaccine..

[27] Li Y, Leneghan DB, Miura K, Nikolaeva D, Brian IJ, Dicks MD (2016). Enhancing immunogenicity and transmission-blocking activity of malaria vaccines by fusing Pfs25 to IMX313 multimerization technology. Sci Rep..

[28] Medley GF, Sinden RE, Fleck S, Billingsley PF, Tirawanchai N, Rodriguez MH (1993). Heterogeneity in patterns of malarial oocyst infections in the mosquito vector. Parasitology.

[29] Churcher TS, Blagborough AM, Delves M, Ramakrishnan C, Kapulu MC, Williams AR (2012). Measuring the blockade of malaria transmission—an analysis of the standard membrane feeding assay. Int J Parasitol.

[30] Delves MJ, Sinden RE (2010). A semi-automated method for counting fluorescent malaria oocysts increases the throughput of transmission blocking studies. Malar J..

[31] Reverberi R, Reverberi L (2007). Factors affecting the antigen-antibody reaction. Blood Transfus..

