# Supplementary material for: An inter-laboratory comparison of standard membrane-feeding assays for evaluation of malaria transmission-blocking vaccines
Source: Malar J. 2016 Sep 9;15(1):463. doi: 10.1186/s12936-016-1515-z (PMC5016893; doi:10.1186/s12936-016-1515-z)
Supplement: Supplementary file 1 — 10.1186/s12936-016-1515-z IC50 calculations for 85RF45.1 mAb. [file 12936_2016_1515_MOESM1_ESM.pdf]

Additional file 1: IC_{50} calculations for 85RF45.1 mAb

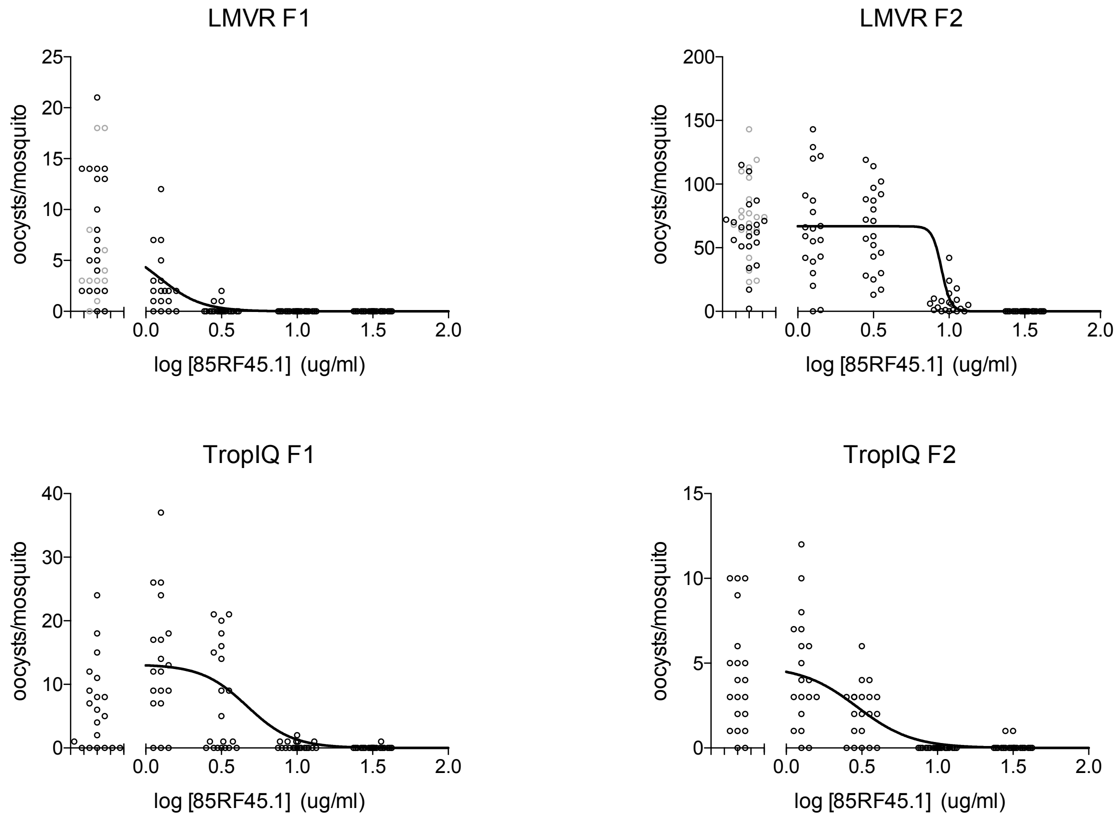

In both laboratories, rat 85RF45.1 mAb was tested at 1.2, 3.5, 10.4 and 31.3 µg/ml in two feeding experiments (F1 and F2). Oocyst count in each mosquito and the best fit to a Hill equation are shown.
