# Supplementary material for: An inter-laboratory comparison of standard membrane-feeding assays for evaluation of malaria transmission-blocking vaccines
Source: Malar J. 2016 Sep 9;15(1):463. doi: 10.1186/s12936-016-1515-z (PMC5016893; doi:10.1186/s12936-016-1515-z)
Supplement: Supplementary file 2 — 10.1186/s12936-016-1515-z IC50 calculations for positive human pAb. [file 12936_2016_1515_MOESM2_ESM.pdf]

Additional file 2: IC_{50} calculations for positive human pAb

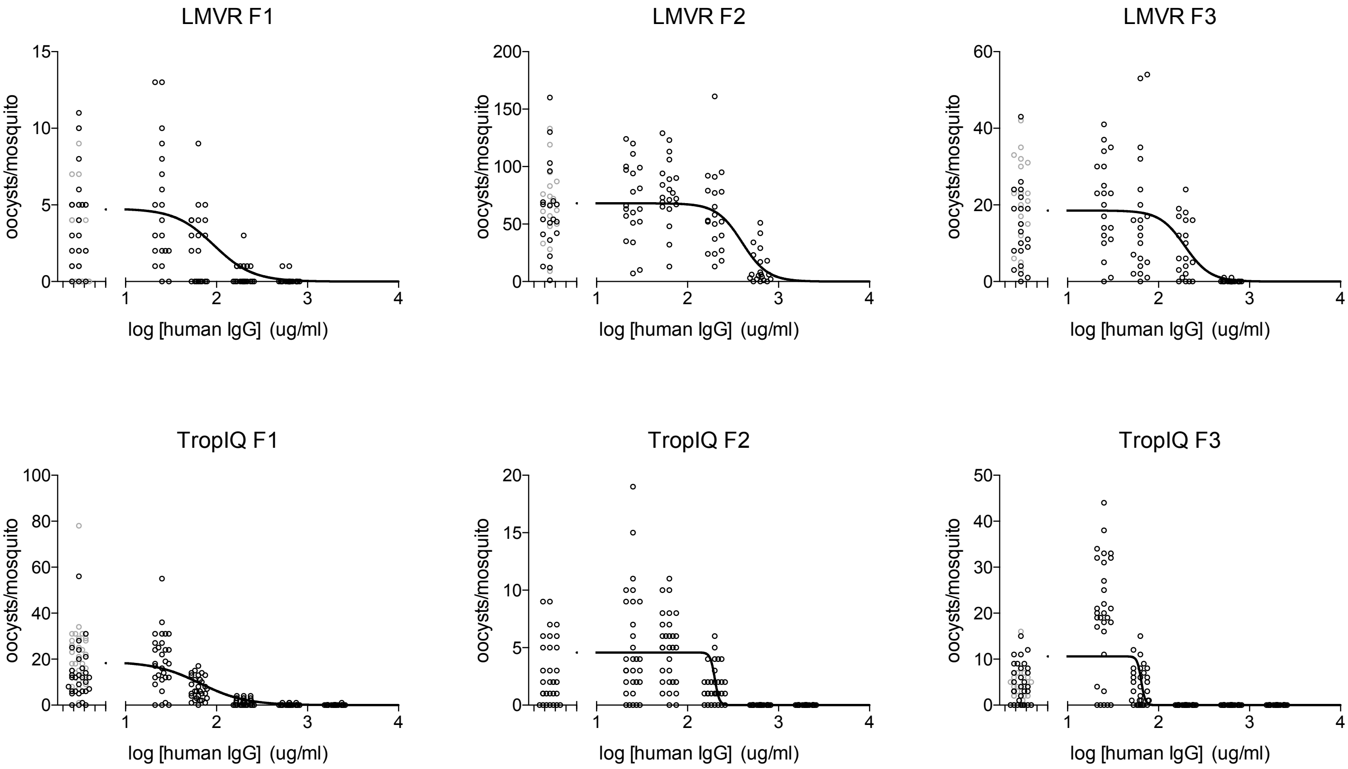

In both laboratories, positive human IgG was tested at 23, 70, 10.4, 210 and 630 µg/ml in three feeding experiments (F1, F2 and F3) . Oocyst count in each mosquito and the best fit to a Hill equation are shown.
